# Evaluation of the population-level impacts of the LiveLighter® obesity prevention campaign from 2012 to 2019 based on serial cross-sectional surveys

**DOI:** 10.1186/s12889-024-18462-5

**Published:** 2024-04-12

**Authors:** Lauren Humphreys, Belinda Morley, Tegan Nuss, Helen Dixon, Gina L. Ambrosini, Ciara O’Flaherty, Melissa Ledger, Ainslie Sartori, Melanie Wakefield

**Affiliations:** 1 WA Department of Health, Perth, WA Australia; 2 Centre for Behavioural Research in Cancer, Melbourne, VIC Australia; 3 Melbourne School of Population and Global Health, The University of Melbourne, Parkville, VIC Australia; 4 Melbourne School of Psychological Sciences, The University of Melbourne, Parkville, VIC Australia; 5 Curtin School of Population Health, Faculty of Health Sciences, Curtin University, Bentley, WA Australia; 6 School of Population and Global Health, University of Western Australia, Crawley, WA Australia; 7 Cancer Council Western Australia, Subiaco, WA Australia

**Keywords:** Mass media campaign, Public health, Nutrition, Obesity prevention, Health behaviours, Healthy weight, Evaluation

## Abstract

**Background:**

Halting and reversing the upward trend in obesity requires sustained implementation of comprehensive, evidence-based strategies at the population-level. The LiveLighter® program targets adults using a range of public education strategies, including mass media campaigns, to support healthy lifestyle changes to attain or maintain a healthy weight and reduce the risk of chronic disease. LiveLighter® has been implemented in Western Australia (WA) since 2012 and, to our knowledge, includes the longest running adult-targeted mass media campaign for healthy weight and lifestyle promotion and education globally. This evaluation assessed the impact of LiveLighter® on WA adults’ knowledge, intentions and behaviours as they relate to healthy eating and body weight from 2012 to 2019.

**Methods:**

LiveLighter® mass media campaigns, which are TV-led and aired statewide, depict genuine, graphic imagery of visceral fat around internal organs to raise awareness about the link between excess body weight and chronic diseases; demonstrate how unhealthy food and drink consumption can contribute to unhealthy weight gain; and recommend healthy alternatives. Cross-sectional telephone surveys were conducted at baseline and following each campaign phase with an independent, randomly selected sample of WA adults aged 25 to 49 years (*n* = 501 to *n* = 1504 per survey) to assess their knowledge of the link between excess body weight and chronic diseases, and their intentions and behaviours related to healthy eating and weight. Multivariable logistic regression models were undertaken to assess differences in responses between baseline and each post-campaign survey.

**Results:**

Compared to baseline, there were significant increases in the proportion of respondents reporting knowledge of excess body weight as a risk factor for certain cancers and type 2 diabetes, intentions to eat more fruit and vegetables and drink less sugar sweetened beverages (SSBs) in the next seven days, and the proportion of respondents who reported meeting guidelines for daily vegetable intake. Reported consumption of SSBs significantly decreased.

**Conclusions:**

LiveLighter® is associated with improvements in knowledge of the health risks associated with excess body mass, increased vegetable intake and reduced SSB consumption in WA adults. These findings support the use of sustained, well-designed healthy lifestyle promotion and education programs as part of a comprehensive obesity prevention strategy.

**Supplementary Information:**

The online version contains supplementary material available at 10.1186/s12889-024-18462-5.

## Background

Globally, obesity is a major public health concern [1–4]. Australia is among a number of high-income, developed countries with a high prevalence of overweight and obesity [5]. In the state of Western Australia (WA), the setting for the present study, nearly three quarters (72%) of the population of 2.7 million people aged 16 years and over are living with either overweight or obesity, placing them at increased risk of serious non-communicable diseases including type 2 diabetes, cardiovascular disease and thirteen types of cancer [6–8]. In 2015-16, 9% ($340 million AUD) of all inpatient hospitalisation costs in WA were attributable to excess body mass [9]. If current trends in overweight and obesity continue, hospital costs in WA are predicted to increase by 80% by 2026 [9].

Halting and reversing the upward trend in obesity prevalence requires sustained implementation of comprehensive, evidence-based strategies at the population-level [1–4, 10–14]. The use of public health campaigns to improve nutrition, increase physical activity and prevent obesity is supported and recommended by peak public health agencies, including the World Health Organization (WHO) [15–17].

Mass media campaigns use mass-reach communication channels such as television and radio to access a large population or population sub-groups, and have been used to protect and promote public health in areas such as tobacco control, road safety, mental health and alcohol harm prevention for decades [18]. Mass media campaigns are recognised as a critical component of a comprehensive best-practice public education program [15, 19]. In Australia, mass media campaigns to improve nutrition, increase physical activity and prevent obesity have been implemented at the state and national level since 2008 [15, 20–27]. Mass media campaigns have proven effective in increasing awareness and knowledge of the benefits of healthy eating and physical activity, and there is some evidence they increase intentions and behaviours as they relate to the adoption of a healthy diet and physical activity behaviours [19, 27].

Mass media campaigns influence behaviour change through a number of different pathways, including increasing awareness of negative health effects, identifying obstacles to change, providing practical advice for change,, and associating positive emotions with change [19]. Theories of health behaviour predict that mass media campaigns may initially influence knowledge, attitudes, beliefs and intentions before impacting behaviour, and that behaviour change can be a long-term process [28–32]. Mass media campaigns targeting health behaviours can also promote discussion about health issues, de-normalise harmful behaviours and encourage public policy changes [19]. For these reasons, sustained delivery of public health mass media campaigns is imperative to achieving and maintaining population-level changes in knowledge, attitudes and intentions, and subsequent health behaviours, which have potential to lead to reductions in population prevalence of overweight and obesity [19].

In response to the increasing prevalence of overweight and obesity, the WA Department of Health commissioned the LiveLighter® healthy weight and lifestyle education and promotion program in 2012. LiveLighter® is a program that targets the adult population (aged 25 to 64 years) using a comprehensive range of public education strategies and mass media campaigns. In recognition that dietary and physical activity behaviours are not simply individually driven, but are strongly shaped by broader environmental, social, economic, and commercial factors, the LiveLighter® program is one component of WA’s comprehensive obesity prevention strategy, which includes working in partnership with other agencies and sectors to influence the social determinants of health for overweight and obesity prevention [33, 34].

The LiveLighter® campaign is informed by the Health Belief Model, the Theory of Planned Behaviour, and rigorous formative research [29, 35, 36]. A range of strategies are used to deliver campaign messages including: depiction of genuine, graphic imagery of visceral fat around the internal organs to raise awareness about the link between excess body weight and chronic diseases [for example, see Additional file 1]; demonstration of how unhealthy food and drink consumption can contribute to unhealthy weight gain and recommendations for healthy alternatives [for example, see Additional file 2 and 3]; and promotion of small, achievable lifestyle changes to attain or maintain a healthy weight and reduce the risk of chronic disease [for example, see Additional file 4 and 5]. Materials from the LiveLighter® program have been licensed for use in six Australian states and territories, and in New York City. To our knowledge, the campaign delivered as part of the LiveLighter® program is the longest running mass media campaign for healthy weight and lifestyle promotion and education targeted to the general adult population in the world [27].

LiveLighter® is evaluated using serial cross-sectional telephone surveys, which have been conducted pre-campaign (baseline) and following each campaign phase. Each time a new campaign is developed and implemented, impact evaluation is conducted using a controlled cohort study. The findings from these controlled cohort studies have been published [37–40].

The aim of this study is to evaluate the population-level impacts of LiveLighter® on WA adults’ knowledge, intentions and behaviours as they relate to healthy diet and weight, between 2012 and 2019 using previously unpublished data collected through the serial cross-sectional telephone surveys. The study also reports on process evaluation measures of campaign reach and frequency [41–43].

## Methods

### Intervention

LiveLighter® uses multiple advertising platforms to deliver healthy lifestyle messages to its target audience (adults aged 25 to 64 years). Television (including regional and Indigenous channels) is the primary platform used to deliver campaign messages, and is supported by messaging across other channels including radio, newspapers, movie theatres, video streaming services, online and social media, digital and outdoor media, public transport, and event sponsorships (including major sporting events). The program includes a range of tools and resources accessible through a dedicated website to support the target audience with adopting healthy behaviour changes, as well as targeted advocacy and research to enable changes in policy to support public health [44].

Each successive LiveLighter® campaign has been carefully developed based on rigorous formative research, including a review of best practice approaches to mass media campaigns promoting healthy weight and lifestyles (see Table 1) [37, 45]. A program of qualitative research was undertaken comprising 12 group discussions with participants from lower socioeconomic backgrounds, and with each group comprising a mix of healthy weight, overweight and obese participants based on self-classification. Two groups were conducted with Indigenous Australians. The program’s overall development and evaluation have been guided by principles of behaviour change, an understanding of the multiple environmental factors that influence the relevant behaviours, and analyses of behaviours targeted by the campaign [32, 37, 46]. The campaign messaging and imagery centred on the serious health consequences associated with overweight and obesity and was designed to elicit strong emotional responses, health communication tactics which have been identified as being among the most persuasive for promoting a healthy lifestyle [47, 48].

**Table 1:** LiveLighter® campaign waves and evaluation phases

Year	Evaluation Phase	Main Ad(s)	Campaign Period (TV ads aired)**	Metro TV TARPs	Regional TV TARPs	Additional TARPs^¥^	Average Weekly TV TARPs^^^	1 + Reach (%)	3 + Reach (%)	Average Frequency	Survey Period	Sample
2012	**Baseline**										**May/Jun**	*N* = 1003
	**Phase 1**	**Toxic Fat**	**24 Jun-04 Aug ** **(6 weeks)**	**1033**	**1045** **(planned)**		**172**	**79**	**69**	**12**	**End Jul/Aug**	*N* = 1002
		**Toxic Fat**	**02 Sep-6 Oct ** **(5 weeks)**	**662**	**825 ** **(planned)**		**132**	**74**	**62**	**10**	**End Sep/Oct**	*N* = 1001
2013		**Toxic Fat**	**21 Jan-5 May** **(11 weeks)**	**1504**	**1750** **(planned)**		**137**	**Not available**	**Not available**	**Not available**	**May/Jun**	*N* = 1504
	**Phase 2**	Sugary Drinks	14 Jul-31 Aug (7 weeks)	1138	865 (planned)		163	82	Not available	13	Cohort study	
		**Sugary Drinks**	**29 Sep-9 Nov** **(6 weeks)**	**957**	**910 ** **(planned)**		**160**	**80**	**Not available**	**11**	**Oct/Nov**	*N* = 508
2014		**Sugary Drinks**	**02 Feb-10 May ** **(9 weeks)**	**2031**	**1700** **(planned)**		**226**	**88**	**Not available**	**22**	**Mar/Apr**	*N* = 501
	**Phase 3**	**Toxic Fat; ** **Sugary Drinks**	**31 Aug-15 Nov ** **(7 weeks)**	**1814**	**1530** **(planned)**		**259**	**80**	**70**	**18**	**Oct/Nov**	*N* = 1003
2015		Toxic Fat; Sugary Drinks	8 Feb-25 Apr (9 weeks)	1473	1050 (planned)		164	81 (Feb-Mar); 64 (Apr)	68 (Feb-Mar); 48 (Apr)	13 (Feb-Mar); 7 (Apr)		
		Toxic Fat; Sugary Drinks	13 Sep-28 Nov (6 weeks)	1295	1170 (planned)		216	84	76	26		
		Eat Brighter LiveLighter	25 Oct-19 Dec (8 weeks)									
2016	**Phase 4a**	**Junk Food: FFO, SS, VM**	**03 Apr-14 May ** **(6 weeks)**	**1064**	**910 ** **(planned)**		**177**	**79**	**65**	**14**	**May/Jun; Cohort study**	*N* = 501
		16 Teaspoons	22 May-31 Dec (20 weeks)		2110 (planned)			Not available	Not available	Not available		
		Junk Food: FFO, SS, VM	19 Jun-9 Jul (3 weeks)	602	620 (planned)		201	68	61	9		
		Junk Food: FFO, SS, VM	28 Aug-1 Oct (4 weeks)	781	640 (planned)		195	72	58	11		
2017	Phase 4b	Junk Food: FFO, SS, VM	26 Feb-22 Apr (8 weeks)	1233	918		154	76	62	14		
		**Junk Food: FFO, SS, VM**	**09 Jul-07 Oct ** **(8 weeks)**	**949**	**610**		**119**	**78** **(Jul-Nov combined)**	**41** **(Jul-Nov combined)**	**18** **(Jul-Nov combined)**	**Oct/Nov**	*N* = 751
		Eat Brighter LiveLighter	29 Oct-9 Dec (4 weeks)									
		Junk Food: FFO, SS, VM	29 Oct-25 Nov (4 weeks)	562	685		141	78 (Jul-Nov combined)	41 (Jul-Nov combined)	18 (Jul-Nov combined)		
2017–2018		Don’t Be Sucked In	26 Nov-17 Feb (12 weeks)									
		Junk Food in Sport	3 Dec-3 Mar (13 weeks)									
2018		Eat Brighter LiveLighter	18 Feb-7 Apr (7 weeks)									
	**Phase 5**	**Sugary Drinks**	**18 Mar-2 June** **(6 weeks)**	**872**	**951**	**174/153 ** **(M/R AFL)** **194/110 ** **(M/R CWG)**	**145**	**69**	**54**	**12**	**May/Jun**	*N* = 751
		Junk Food: SS	8 Jul-3 Sep (6 weeks)	1588	1100	124/149 (M/R AFL)	264	75	64	18		
		Eat Brighter LiveLighter	30 Sep-10 Nov (6 weeks)									
		Sugary Drinks	23 Sep-1 Dec (6 weeks)	834	747	24/13 (M/R AFL)	139	66	49	12		
		Don’t Be Sucked In	25 Nov-16 Feb (12 weeks)									
		Junk Food in Sport	2 Dec-16 Feb (11 weeks)									
2019		Eat Brighter LiveLighter	17 Feb-13 Apr (8 weeks)									
	**Phase 6**	**Junk Food: FFO, SS, VM**	**28 Apr-22 Jun ** **(6 weeks)**	**915**	**631**	**296/288** **(M/R AFL)**	**153**	**66**	**52**	**13**	**Jun/Jul**	*N* = 1004

* Eat Brighter LiveLighter, Don’t Be Sucked In, and Junk Food in Sport were non-television campaigns

¥ Additional TARPs includes TARPs achieved outside of the main commercial television media buy (e.g., Australian Football League (AFL) and Commonwealth Games (CWG) broadcasts). M/R = Metro/Regional.

^ From 2012–2016 planned regional TARPs are reported because achieved regional TARPs were not available. Weekly average TARPs were therefore calculated using metro TARPs only and the number of weeks the campaign aired on metro channels

† Reach is presented as the percentage of the target audience exposed to each advertisement at least once (1 + reach) and at least three times (3 + reach)

Notes. The Junk Food campaign included three advertisements: Fast Food Outlet (FFO), Service Station (SS), Vending Machine (VM). In 2018, only the Service Station advertisement was aired as part of the Junk Food campaign

The LiveLighter® program was launched in June 2012, with Phase 1 ‘Toxic Fat 2012/13’ of the mass media campaign. Phase 1 included three waves of the ‘Toxic Fat’ television advertisement, which shows graphic real life footage of visceral fat around the organs of a person with overweight and describes the link between visceral fat and chronic disease, as a way of communicating increased urgency to change behaviour. Phase 2 ‘Sugary Drinks 2013/14’ of the mass media campaign launched in 2013 and included three waves of the ‘Sugary Drinks’ television advertisement, which reminds viewers of the visceral ‘toxic fat’ imagery and focusses on the contribution of sugary drink consumption to the development of ‘toxic fat’ and an increased risk of disease. Phase 3 ‘Toxic Fat/Sugary Drinks 2014’ of the campaign combined the ‘Toxic Fat’ and ‘Sugary Drinks’ advertisements in three waves of mass media.

Phase 4a ‘Junk Food 2016’ of the campaign comprised three waves of the ‘Junk Food’ television advertisements delivered in 2016. A further two waves of the ‘Junk Food’ advertisements were delivered in 2017 as part of Phase 4b ‘Junk Food 2017’. The ‘Junk Food’ campaign comprised three advertisements ‘Fast Food Outlet’, ‘Service Station’ and ‘Vending Machine’, which depict the same ‘toxic fat’ imagery and focus on the contribution of consumption of junk food (including fast food, sweet foods, salty snacks) to the development of ‘toxic fat’ and disease. Phases 4a and 4b also included a new message about the link between overweight and fatty liver disease. In 2018, Phase 5 ‘Sugary Drinks 2018’ of the campaign centred again on the ‘Sugary Drinks’ television advertisement, first run in 2013. In 2019, Phase 6 ‘Junk Food 2019’ of the campaign ran the ‘Junk Food’ advertisements again.

### Process evaluation

Advertisements that achieve higher reach and frequency are more likely to be recalled by the target audience [41]. Reach and frequency are important determinants of a campaign’s ability to have an impact on the intended target audience [42, 43]. Advertising exposure data were collected on reach, frequency and Target Audience Rating Points (TARPs). Reach is presented as the percentage of the target audience exposed to each advertisement at least once (1 + reach) and at least three times (3 + reach). Frequency is presented as the average number of times a target audience member is exposed to an advertisement. TARPs are calculated as the product of ‘1 + reach’ and ‘frequency’, and these figures are cumulated over time [37, 49]. For example, 200 TARPs per week may represent 100% of the target audience being exposed to the message an average of two times or 50% reached four times [37].

### Impact evaluation

#### Evaluation design and sample

To evaluate the impacts of the LiveLighter® campaign, a series of cross-sectional telephone surveys were conducted with a representative sample of adults aged 25 to 49 years who resided in private households in metropolitan and regional areas of WA. Table 1 provides an overview of the evaluation surveys relative to the timing of each phase of LiveLighter®. Survey phases were timed to coincide with the final two weeks of the campaign period for consistency and recency to aid campaign recall. Data collection was conducted by the Survey Research Centre at Edith Cowan University using Computer Assisted Telephone Interviews (CATI).

From baseline (2012) through to Phase 5 ‘Sugary Drinks 2018’, a Random Digit Dialling (RDD) sample frame of landline household phone numbers was used. Data collected in Phase 6 (‘Junk Food 2019’) used a blended sampling frame. The sample frame comprised a 50% sample of RDD landline household phone numbers, and 50% database mobile phone numbers and landline household phone numbers (85% mobile, 15% landline) sample to address changing telephone use in Australia [50]. Analysis of the samples indicated the demographic profile of the blended sample was comparable to past surveys [51]. Quotas were set at 35% for the 25 to 34 age group, with soft quotas relating to gender (male 50%, female 50%) and region (metropolitan 70%, regional 30%) in order to ensure the samples were representative of the WA population. Survey response rates ranged from 19 to 44% across the evaluations of each campaign phase.

### Measures

Each post-campaign survey assessed respondents’ awareness of the advertisements shown at each campaign wave, knowledge of the link between excess body weight and chronic diseases, and their intentions and behaviours as they relate to healthy weight and diet.

#### Campaign awareness

To measure campaign awareness, both unprompted recall and prompted recognition of the advertisements shown at each campaign phase were assessed. Unprompted recall was assessed by asking respondents if they had seen advertising about being overweight, and then if they answered yes, asking them to describe the advertisement(s) they had seen. These descriptions were then coded by at least two authors to determine whether respondents were describing the LiveLighter® advertisement(s) shown during each campaign phase. Discrepancies in coding were discussed by the authors until consensus was attained. Prompted recognition was assessed by describing the video advertisement(s) to respondents and asking whether they had seen the ad(s) on TV (including catch up TV) or online.

#### Knowledge, intentions and behaviours

Questionnaire wording, response options and binary aggregation for analysis are given in Table 2 for outcomes relating to knowledge, intentions and behaviours. The survey instrument was pilot tested before administration. Knowledge of the link between excess body weight and chronic diseases was measured by assessing respondents’ perceived likelihood that being overweight is a risk factor for heart disease, type 2 diabetes or cancer. Perceived likelihood was measured using a five-point Likert scale from ‘Very likely’, ‘Slightly unlikely’, ‘Neither unlikely nor likely’, ‘Slightly likely’ to ‘Very unlikely’.

**Table 2:** Outcome measures: questionnaire wording, response options and binary aggregation for analysis

Outcome	Question	Response options	Binary aggregation
**Knowledge of the link between overweight and chronic disease** ^**a**^			
**Knowledge of link between overweight and heart disease**	How likely do you think being overweight is a risk factor for… heart disease?	Very unlikely; Slightly unlikely; Neither unlikely nor likely; Slightly likely; Very likely; (Don’t know); (Refused).	Very/Slightly likely cf. all other responses^b^.
**Knowledge of link between overweight and type 2 dabetes**	How likely do you think being overweight is a risk factor for… type 2 diabetes?		
**Knowledge of link between overweight and cancer**	How likely do you think being overweight is a risk factor for… cancer?		
***Prompted dietary intentions*** ^**a**^			
**Likely to cut down amount of high calorie food in next 7 days**	Over the next 7 days, how likely or unlikely are you to… cut down the amount of high calorie food you eat?	Very unlikely; Slightly unlikely; Neither unlikely nor likely; Slightly likely; Very likely; (Don’t know); (Refused).	Very/Slightly likely cf. all other responses^b^.
**Likely to drink less sugary drinks in next 7 days**	Over the next 7 days, how likely or unlikely are you to… drink less sugary drinks?		
**Likely to eat smaller serving sizes in next 7 days**	Over the next 7 days, how likely or unlikely are you to… eat smaller serving sizes?		
**Likely to eat more fruit and vegetables in next 7 days**	Over the next 7 days, how likely or unlikely are you to… eat more fruit and vegetables?		
***Behaviour***			
**Fruit consumption**	Thinking back over the past 7 days, how many serves of fruit did you usually eat each day? A serve of fruit is equal to one medium piece, two small pieces of fruit or one cup of diced fruit.	Serves per day; None; Less than one a day; (Don’t know); (Refused).	2 or more serves a day cf. all other responses^b^.
**Vegetable consumption**	Thinking back over the past 7 days, how many serves of vegetables did you usually eat each day? A serve of vegetables is equal to half a cup of cooked vegetables or 1 cup of salad.	Serves per day; None; Less than one a day; (Don’t know); (Refused).	For females: 5 or more serves a day cf. all other responses^b^. For males: 6 or more serves day cf. all other responses^b^
**Sugar sweetened beverage (SSB) consumption**	(a) During the past 7 days, on how many days did you drink a can, bottle or glass of a sugar-sweetened drink such as soft drinks, energy drinks, fruit drink, sports drinks and cordial? Do not include diet drinks. (Interviewer note: fruit drink does not include 100% fruit juice). IF 1 to 7: (b) On days that you did drink sugar-sweetened drinks, how many times per day did you usually drink them?	(a) Days in the past 7 drank SSB (Range 0–7); (Don’t know); (Refused); (b) Once a day; twice a day; 3 times per day; 4 or more times per day; (Don’t know); (Refused).	1 or more times in last week cf. all others; 4 or more times in last week cf. all other responses^b^.
**Fast food consumption**	(a) During the past 7 days, on how many days did you eat take-away or ‘fast foods’ (such as fish and chips, hamburgers, fried chicken, pizza, sausage rolls, meat pies)? IF 1 to 7: (b) On days that you did eat take-away or ‘fast food’, how many times per day did you usually eat it?	(a) Days in the past 7 ate fast food (Range 0–7); (Don’t know); (Refused); (b) Once a day; twice a day; 3 times per day; 4 or more times per day; (Don’t know); (Refused).	1 or more times a week cf. all other responses^b^.
**Sweet food consumption**	(a) During the past 7 days, on how many days did you eat sweet foods (such as cakes, biscuits, lollies and chocolates)? IF 1 to 7: (b) On days that you did eat sweet foods, how many times per day did you usually eat it?	a) Days in the past 7 ate sweet foods (Range 0–7); (Don’t know); (Refused); b) Once a day; twice a day; 3 times per day; 4 or more times per day; (Don’t know); (Refused).	3 or more times a week cf. all other responses^b^.

^a^ Presentation of questions in this set was randomised to avoid potential order effects. Single responses only were allowed

^b^ Responses of ‘Don’t Know’ and ‘Refused’ were excluded

Intentions to change behaviour, including cutting down on high calorie food, drinking less sugar-sweetened beverages (SSBs), eating smaller serving sizes, and eating more fruit and vegetables in the next seven days from the time respondents were interviewed, were also assessed using perceived likelihood.

For dietary behaviours, respondents were asked about their frequency of consumption of fruit, vegetables, SSBs, fast food and sweet foods over the past seven days using questions based on Australian national nutrition surveys [52, 53]. A description of a serving of fruit and a serving of vegetables, as defined by the Australian Dietary Guidelines (ADG), was provided to respondents in the telephone survey to aid understanding [54]. Proportions were then calculated to determine the percentage of respondents who were meeting ADG recommendations for fruit intake (at least two serves per day) and vegetable intake (at least five serves per day for females and at least six serves per day for males) [55], SSBs one or more times per week, SSBs four or more times per week, fast food one or more times per week, and sweet food three or more times per week.

#### Sociodemographic characteristics

Data were collected on respondents’ sociodemographic characteristics, including gender, age, parental status (a parent or guardian to a child under 18), educational attainment, and whether respondents indicated they were of Aboriginal or Torres Strait Islander descent. Respondents’ residential postcodes were used to determine geographical location (i.e., metropolitan or regional). Postcode was also used to determine socioeconomic area according to the Index of Relative Socio-Economic Disadvantage (IRSD) rankings for WA, which identifies and ranks areas in terms of their relative socio-economic disadvantage, with low IRSD indicating greater disadvantage and high IRSD indicating least disadvantage [56]. Respondents’ self-reported height and weight was used to calculate their body mass index (BMI), which was classified into weight categories (< 25 kg/m^2^ for ‘not overweight or obese’, and ≥ 25 kg/m^2^ for ‘overweight or obese’) according to internationally recognised cut-offs [57]. Respondents were also asked about how many hours they would normally spend watching commercial TV on an average weekday. A dichotomous variable was created to indicate the proportion watching for two or more hours per day, based on OzTAM and Nielsen data showing the average commercial TV viewing time per day for Australian adults [58].

### Statistical analyses

Data were analysed using StataMP 16.0 [59] and weighted by age, gender, location and educational attainment to ensure the samples were representative of the WA population [56]. Surveys phases baseline (2012) through to Phase 4b ‘Junk Food 2017’ were weighted using 2011 Census data, while Phase 5 ‘Sugary Drinks 2018’ and Phase 6 ‘Junk Food 2019’ were weighted using 2016 Census data. Weighted proportions and unweighted samples (N) are reported. A significance level of *p* < 0.05 was applied throughout. Chi-square tests for independence were used to examine differences in demographic characteristics across study phases. Since there were significant differences by study phase in the proportion of respondents across BMI category, socioeconomic area, parental status, having completed some tertiary education, and reporting watching commercial television for two or more hours per day, these variables were included as covariates in subsequent multivariable logistic regression models. Logistic regression models examining intentions and behaviours relating to sugary drink consumption also controlled for seasonality (i.e., based on survey period). People are motivated to drink more in order to stay hydrated and industry advertising expenditure for sugary drinks has been shown to peak during warmer months (i.e., Mar/Apr, Sep/Oct, Oct/Nov) compared to cooler months (i.e., May/Jun, Jun/Jul, Jul/Aug) [60, 61].

Responses for all knowledge, intentions and behaviour outcomes were collapsed to form binary variables. As the proportion of ‘Don’t know’ or ‘Refused’ responses was very small for each variable (< 1.0–1.1% for all except knowledge of the link between overweight and cancer which was 6.6% overall), it was not possible to analyse whether there were changes in these responses over time and so all analyses excluded responses of ‘Don’t know’ or ‘Refused’. Multivariable logistic regression models were undertaken to assess differences in the dependent variables (i.e., knowledge, intentions and behaviours) by the independent variable (i.e., campaign phase). Baseline (pre-campaign) was used as the reference category to assess any differences at subsequent study phases.

## Results

Table 3 presents survey sample characteristics by study phases, from baseline (2012) to Phase 6 ‘Junk Food 2019’. Across study phases, the survey samples had comparable profiles in terms of gender, age group, residential location, and whether respondents indicated they were of Aboriginal or Torres Strait Islander descent.

**Table 3:** Sample characteristic proportions, by study phase

	Baseline 2012	Phase 1 Toxic Fat 2012/13	Phase 2 Sugary Drinks 2013/14	Phase 3 Toxic Fat/ Sugary Drinks 2014	Phase 4a Junk Food 2016	Phase 4b Junk Food 2017	Phase 5 Sugary Drinks 2018	Phase 6 Junk Food 2019	WA Population 2016
	*n* = 1003	*n* = 3507	*n* = 1009	*n* = 1003	*n* = 501	*n* = 751	*n* = 751	*n* = 1004	
**Gender**									
Male	45.1	45.0	44.8	44.0	43.7	46.5	45.4	45.4	50.0
Female	54.9	55.0	55.2	56.0	56.3	53.5	54.6	54.6	50.0
**Age**									
25–34 years	34.9	34.8	34.7	34.1	33.7	34.6	34.6	34.9	42.3^d^
35–44 years	40.4	39.3	40.2	39.1	37.7	35.0	34.1	37.8	38.5^d^
45 + years	24.7	25.9	25.1	26.8	28.5	30.4	31.3	27.4	19.2^d^
**Body Mass Index** ^**a**^ *									
Not overweight/obese	44.5	46.2	46.7	43.5	45.4	41.5	43.8	40.3	35.0^e^
Overweight/obese	55.5	53.8	53.3	56.5	54.6	58.5	56.2	59.8	65.0^e^
**Residential Location**									
Metropolitan	70.0	70.0	69.6	70.0	70.1	69.8	69.9	69.8	74.8
Regional	30.0	30.0	30.4	30.0	29.9	30.2	30.1	30.2	25.2
**Socio Economic Area** ^**b**^ *									
Low	32.8	32.7	30.5	35.1	33.9	28.1	31.8	32.4	34.5
Mid	36.1	36.8	38.9	35.8	32.9	38.7	36.2	44.3	37.8
High	31.2	30.6	30.7	29.2	33.3	33.2	32.0	23.3	27.7
**Parent** ^**c**^ *									
Yes	57.5	60.1	64.3	62.5	54.1	55.8	63.7	64.6	N/A
Education *									
Some tertiary education or above^f^	69.6	70.2	71.8	75.7	73.8	79.0	80.0	76.7	46.5
**Commercial TV viewing** *									
More than 2 h/day	19.5	13.6	10.8	9.5	13.0	12.7	9.8	8.4	N/A
**Aboriginal or Torres Strait Islander Descent**									
Yes	1.6	1.2	2.1	1.6	2.0	0.9	2.0	1.9	3.1

Note: Unweighted percentages. Percentages are rounded so may not sum to 100%

*Significant difference across phase (*p* < 0.05)

^a^ Adults’ self-reported height and weight was used to calculate their BMI: weight (kg) / [height (m)] [2] which was classified into weight categories according to internationally recognised cut-offs [75]. BMI could not be calculated for *n* = 30 at baseline, *n* = 95 at Toxic Fat 2012/13, *n* = 24 at Sugary Drinks 2013/14, *n* = 32 at Toxic Fat/Sugary Drinks 2014, *n* = 16 at Junk Food 2016, *n* = 19 at Junk Food 2017, *n* = 22 at Sugary Drinks 2018, and *n* = 25 at Junk Food 2019 due to missing data

^b^ Socioeconomic area was determined according to the Index of Relative Socio-Economic Disadvantage (IRSD) rankings for Western Australia as described by the Australian Bureau of Statistics (2016) [56] based on respondents’ home postcodes. Low IRSD indicates greater disadvantage, high IRSD indicates least disadvantage. No IRSD value was available (or unknown/invalid postcode reported) for *n* = 5 at baseline 2012, *n* = 20 at Toxic Fat 2012/13, *n* = 4 at Sugary Drinks 2013/14, *n* = 5 at Toxic Fat/Sugary Drinks 2014, *n* = 5 at Junk Food 2016, *n* = 7 at Junk Food 2017, *n* = 5 at Sugary Drinks 2018, and *n* = 7 at Junk Food 2019

^c^ Respondents were classified as parents if they indicated they were the parent or main carer of one or more children aged under 18 who live with them

^d^ WA population proportions for these age groups were calculated as proportions of this age range (25–49 years) in the general population

^e^ Source: WA Health and Wellbeing Surveillance System (2016)

^f^ Tertiary education is defined as formal post-secondary education, including qualifications ranging from undergraduate awards (bachelor’s degrees, associate degrees and advanced diplomas) to postgraduate awards (graduate certificates and diplomas, master’s and doctoral degrees)

### Process evaluation

The number of TARPs and other process evaluation measures such as frequency of TV advertisement exposure (1 + reach, 3 + reach and average frequency) indicated a high level of campaign reach across metropolitan and regional areas of WA, with more than two thirds of the target audience consistently being exposed to the advertisements more than once (see Table 1).

### Impact evaluation

**Fig. 1:**
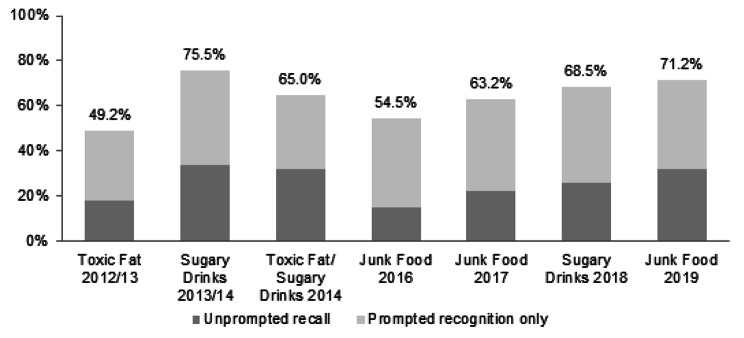
Campaign awareness (unprompted recall + prompted recognition only) of the LiveLighter^®^ advertisements, by study phase. Note. Not measured at Baseline. The data reported here is total awareness (unprompted recall plus prompted recognition only) of the specific LiveLighter^®^ ad(s) shown at each phase.Base. All respondents (*N* = 8,526)

Figure 1 shows the proportion of respondents who were aware of the LiveLighter® advertisement(s) at each phase. Total awareness was highest following the first campaign focussed on SSB consumption (Phase 2 ‘Sugary Drinks 2013/14’: 75.5%) and next highest for the most recent SSB and junk food campaigns (Phase 5 ‘Sugary Drinks 2018’: 68.5%; Phase 6 ‘Junk Food 2019’: 71.2%). Awareness was lowest at the first phase of LiveLighter® (Phase 1 ‘Toxic Fat 2012/13’: 49.2%).

Table 4 presents the proportions for knowledge, intentions and behaviours by campaign phase and the results of the multivariable logistic regression analyses (Adjusted Odds Ratios [AORs] and 95% CIs) comparing baseline to each subsequent phase.

**Table 4:** Knowledge, intentions and behaviour, by study phase

	Baseline	Phase 1	Phase 2	Phase 3	Phase 4a	Phase 4b	Phase 5	Phase 6
Outcome	2012	Toxic Fat 2012/13	Sugary Drinks 2013/14	Toxic Fat/ Sugary Drinks 2014	Junk Food 2016	Junk Food 2017	Sugary Drinks 2018	Junk Food 2019
	**%**	**% ** (AOR; 95% CI)	**% ** (AOR; 95% CI)	**% ** (AOR; 95% CI)	**% ** (AOR; 95% CI)	**% ** (AOR; 95% CI)	**% ** (AOR; 95% CI)	**% ** (AOR; 95% CI)
*Knowledge of the link between overweight and chronic disease*								
Knowledge of link between overweight and heart disease	88.0^†^	90.8* **(1.38; 1.03–1.85)**	90.6 (1.4; 0.96–2.06)	87.9 (1.07; 0.75–1.55)	89.7 (1.32; 0.84–2.08)	86.7 (0.89; 0.62–1.29)	85.9 (0.82; 0.57–1.20)	90.5 (1.24; 0.89–1.74)
Knowledge of link between overweight and type 2 diabetes	84.3^†^	89.5* **(1.58; 1.21–2.08)**	91.7* **(2.24; 1.54–3.28)**	86.5 (1.30; 0.92–1.82)	91.3* **(2.30; 1.43–3.69)**	89.5* **(1.53; 1.05–2.22)**	86.8 (1.23; 0.85–1.80)	89.4* **(1.53; 1.11–2.11)**
Knowledge of link between overweight and cancer	40.9^†^	52.0* **(1.59; 1.32–1.92)**	65.9* **(2.83; 2.22–3.60)**	54.5* **(1.78; 1.40–2.25)**	55.6* **(1.84; 1.39–2.45)**	49.0* **(1.38; 1.07–1.77)**	58.7* **(2.12; 1.64–2.74)**	55.2* **(1.74; 1.39–2.17)**
*Prompted dietary intentions*								
Likely to cut down amount of high calorie food in next 7 days	52.0^†^	57.4* **(1.24; 1.03–1.49)**	53.3 (1.03; 0.82–1.29)	49.6 (0.87; 0.69–1.10)	45.4 (0.78; 0.59–1.03)	52.0 (0.99; 0.78–1.26)	46.4* **(0.76; 0.59–0.97)**	53.3 (1.05; 0.85–1.30)
Likely to drink less sugary drinks in next 7 days	41.8^†^	46.6* **(1.25; 1.03–1.52)**	35.1 (0.74; 0.55–1.02)	35.3 (0.76; 0.56–1.03)	41.5 (1.00; 0.76–1.33)	44.7 (1.11; 0.81–1.52)	38.1 (0.89; 0.69–1.15)	47.1* **(1.30; 1.05–1.61)**
Likely to eat smaller serving sizes in next 7 days	47.8^†^	50.8 (1.13; 0.94–1.36)	47.2 (0.95; 0.76–1.20)	44.4 (0.83; 0.66–1.05)	46.0 (0.96; 0.73–1.28)	42.9* **(0.80; 0.62–1.02)**	38.6* **(0.64; 0.50–0.83)**	46.4 (0.92; 0.74–1.14)
Likely to eat more fruit and vegetables in next 7 days	57.3^†^	62.6* **(1.32; 1.10–1.59)**	59.5 (1.15; 0.91–1.44)	55.9 (0.99; 0.79–1.25)	55.9 (1.03; 0.78–1.36)	61.7* **(1.30; 1.02–1.65)**	60.3 (1.19; 0.93–1.53)	67.6* **(1.75; 1.40–2.18)**
*Dietary behaviours*								
Met ADG recommendation for daily fruit consumption (i.e., at least 2 serves)	49.0^†^	51.0 (1.07; 0.89–1.28)	51.6 (1.07; 0.85–1.34)	49.2 (0.99; 0.79–1.24)	49.4 (1.00; 0.76–1.31)	53.3 (1.18; 0.93–1.50)	48.9 (0.93; 0.73–1.19)	46.3 (0.87; 0.70–1.07)
Met ADG recommendation for daily vegetable consumption (i.e., at least 5 serves for females, at least 6 serves for males)	8.2^†^	8.2 (1.08; 0.79–1.49)	6.4 (0.77; 0.52–1.15)	5.7 (0.74; 0.49–1.12)	7.9 (1.02; 0.63–1.66)	12.2* **(1.66; 1.15–2.40)**	10.9 (1.37; 0.93–2.02)	11.9* **(1.47; 1.04–2.08)**
Consumed sugar sweetened beverages 1 or more times per week	60.2^†^	58.0 (0.92; 0.76–1.12)	54.2 (0.81; 0.59–1.09)	56.5 (0.85; 0.62–1.16)	46.0* **(0.58; 0.44–0.77)**	51.0* **(0.70; 0.51–0.96)**	43.1* **(0.50; 0.39–0.65)**	41.3* **(0.48; 0.39–0.60)**
Consumed sugar sweetened beverages 4 or more times per week	28.3^†^	29.3 (0.94; 0.75–1.17)	27.4 (0.79; 0.56–1.11)	26.3* **(0.67; 0.47–0.95)**	19.2* **(0.60; 0.42–0.84)**	16.0* **(0.38; 0.26–0.56)**	12.1* **(0.35; 0.25–0.50)**	13.8* **(0.43; 0.32–0.56)**
Consumed fast food 1 or more times per week	61.8^†^	62.7 (1.00; 0.83–1.21)	63.3 (1.04; 0.83–1.32)	65.3 (1.18; 0.92–1.49)	54.0* **(0.75; 0.57-1.00)**	61.1 (0.96; 0.75–1.23)	61.8 (1.03; 0.80–1.32)	65.8 (1.21; 0.96–1.51)
Consumed sweet foods 3 or more times per week	49.9^†^	48.5 (0.91; 0.75–1.10)	49.1 (0.92; 0.73–1.17)	46.3 (0.85; 0.67–1.07)	47.5 (0.89; 0.67–1.17)	52.0 (1.02; 0.80–1.30)	45.9 (0.84; 0.65–1.07)	47.6 (0.87; 0.70–1.08)

AOR = Adjusted odds ratio; 95% CI = 95% confidence interval

*Significant difference compared to baseline (†) at *p* < 0.05

### Knowledge of the link between excess body weight and chronic disease

Following commencement of the LiveLighter® campaign, there was a statistically significant increase in knowledge of excess body weight as a risk factor for cancer from baseline to Phase 1 ‘Toxic Fat 2012/13’ (40.9% cf. 52.0%). This was maintained across all phases to Phase 6 ‘Junk Food 2019’ (55.2%). Knowledge of the link between excess body weight and heart disease also increased significantly from baseline to Phase 1 ‘Toxic Fat 2012/13’ (88.0% cf. 90.8%). However, this increase was not maintained across subsequent phases. Knowledge of the link between excess body weight and type 2 diabetes increased significantly from baseline to Phase 1 ‘Toxic Fat 2012/13’ (84.3% cf. 89.5%). This increase was sustained across almost all subsequent phases to Phase 6 ‘Junk Food 2019’.

### Fruit and vegetable consumption intentions and behaviour

There was a statistically significant increase in reported intentions to eat more fruit and vegetables in the next seven days, between baseline and Phase 1 ‘Toxic Fat 2012/13’ (57.3% cf. 62.6%). However, intentions then reverted to baseline levels until Phase 4a ‘Junk Food 2016’, before significantly increasing again following the Phase 4b ‘Junk Food 2017’ campaign (61.7%) and Phase 6 ‘Junk Food 2019’ campaign (67.6%).

Respondents meeting ADG recommendations for daily vegetable intake increased significantly from baseline (8.2%) to Phase 4b ‘Junk Food 2017’ (12.2%) and Phase 6 ‘Junk Food 2019’ (11.9%). Whereas, the reported consumption of at least two serves of fruit per day, in line with ADG recommendations, remained stable from baseline to Phase 6 ‘Junk Food 2019’ (49.0% cf. 46.3%).

### Discretionary food consumption intentions and behaviour

Those respondents intending to drink less sugary drinks in the next seven days increased significantly from baseline to Phase 1 ‘Toxic Fat 2012/13’ (41.8% cf. 46.6%). Intentions returned to baseline levels in subsequent phases before significantly increasing following the Phase 6 ‘Junk Food 2019’ campaign (47.1%).

There was a considerable decrease in respondents reporting consuming SSBs at least once per week following Phase 4a ‘Junk Food 2016’ compared to baseline (60.2% cf. 46.0%). This decrease was maintained across phases to Phase 6 ‘Junk Food 2019’ (41.3%).

Respondents reporting consuming SSBs at least four times per week (classified as ‘heavy consumers’) decreased significantly from baseline following the Phase 3 ‘Toxic Fat/Sugary Drinks 2014’ campaign (28.3% cf. 26.3%). This decrease was maintained across subsequent campaign phases. Following Phase 5 ‘Sugary Drinks 2018’ and Phase 6 ‘Junk Food 2019’, the proportion of respondents classified as heavy consumers halved relative to baseline (12.1% and 13.8%, respectively cf. 28.3%).

Intentions to reduce high calorie foods in the next seven days remained steady from baseline to Phase 6 ‘Junk Food 2019’ despite a significant increase following Phase 1 ‘Toxic Fat 2012/13’ (57.4%) and a significant decline following Phase 5 ‘Sugary Drinks 2018’ (46.4%).

Intentions to eat smaller serving sizes in the next seven days decreased significantly from baseline (47.8%) following the Phase 4b ‘Junk Food 2017’ (42.9%) and Phase 5 ‘Sugary Drinks 2018’ (38.6%) campaigns before returning to baseline levels in subsequent phases.

The cross-sectional data indicated respondents consuming sweet foods three or more times per week remained stable from baseline to Phase 6 ‘Junk Food 2019’ (49.9% cf. 47.6%).

Those who consumed fast food at least weekly decreased significantly from baseline, following the Phase 4a ‘Junk Food 2016’ campaign (61.8% cf. 54.0%). Fast food consumption returned to baseline levels in subsequent campaign phases.

## Discussion

Between 2012 and 2019, positive changes in self-reported knowledge, intentions, and behaviours were observed in surveys of the target population, in line with the aims of the LiveLighter® program to raise awareness about the link between excess body weight and chronic diseases and promote healthy lifestyle changes to attain or maintain a healthy weight and reduce the risk of chronic disease.

A critical success factor with any mass media campaign is audience reach. Research on the use of mass media campaigns in tobacco control suggests that a minimum of 100 weekly TARPs is needed to promote behaviour change, with higher TARPs resulting in more favourable outcomes [39, 62], and behavioural impacts decaying rapidly when campaigns are de-funded, suggesting sustained funding is needed to optimise campaign effects [63, 64]. Despite the significant amount of unhealthy food and drink marketing faced by consumers in their day-to-day lives [65, 66] the LiveLighter® campaign had a strong reach, with weekly TARPS of between 119 and 264 and advertisements reaching between 66 and 88% of the target audience at least once [39, 62]. Strong reach is reflected in the high levels of population awareness associated with the various LiveLighter® campaigns (49–76%). This is supported by an international study on consumer recall of government healthy eating campaigns in five countries: Australia, Canada, Mexico, the United Kingdom and the United States, found that the LiveLighter® campaign had the highest level of recall out of several Australian campaigns and was among the top campaigns for recall internationally [41]. This demonstrates the mass media campaign’s ability to cut-through an increasingly cluttered media environment [41]. Continuing to achieve high reach in an ever more fragmented media market of different channels and platforms requires the ongoing attention of campaign planners and media buyers, as well as evaluators to monitor campaign outcomes.

A central theme of many LiveLighter® campaigns is the use of consistent messaging focussing on the health risks associated with unhealthy dietary habits such as SSB and junk food consumption, in particular the lesser known link between these behaviours, overweight and cancer. This is accompanied by graphic imagery of visceral fat around the organs. This strategy may have contributed to the improvements in knowledge of the link between excess body weight and an increased risk of cancer, which were maintained across all phases of the campaign, and intentions and behaviours as they relate to reducing intake of SSBs and fast food, observed in this study.

Previous population-based evaluations of successive LiveLighter® campaigns and an experimental study testing psychological responses to LiveLighter® advertisements have tested for potential unintended consequences of the campaign, in response to concerns that the campaign could unintentionally stigmatise people with overweight or obesity. Findings indicate the campaign has not had negative psychological or behavioural consequences, such as increased endorsement of weight-based stereotypes, internalised weight bias or maladaptive dietary behaviours [37–39, 67].

In order to effectively promote healthy behaviour change, LiveLighter® campaign messages have a strategic focus on small, achievable lifestyle changes [19, 68]. One example of this is reducing SSBs intake, as SSBs provide no essential nutrients, healthier options are readily available, and avoiding SSBs may involve fewer barriers than other dietary changes [38]. This evaluation found significant increases in intentions to drink less sugary drinks, which were substantiated by reported decreases in SSB consumption, resulting in fewer people being classified as moderate and heavy sugary drink consumers. The greatest decreases were observed for heavy consumers of sugary drinks (consumption of SSBs 4 or more times per week). Clear messaging focussing on avoiding sugary drinks as well as the graphic evidence illustrating their potential health effects, may have contributed towards these positive changes in SSB consumption [38].

Research has shown that programs applying a comprehensive approach have the greatest impact [27, 69]. The LiveLighter® program includes messaging on the broader determinants of obesity and dietary habits, including common situational enablers (for example, the availability of healthy options for food and drink in shopping centre food courts) and barriers to healthy behaviour change (for example, advertising and promotion of unhealthy food and drink in supermarkets) to complement the program’s behaviour change messages. Consistent with evidence on best practice messaging in public health education campaigns, LiveLighter® advertisements frequently depict common scenarios where a person is presented with an unhealthy option when undertaking usual daily activities, with a call to action to avoid the unhealthy option altogether or select a healthy alternative [19, 20, 69]. For example, the ‘Junk Food’ campaign shows how the availability and promotion of unhealthy snacks and drinks at the point of purchase at petrol stations, vending machines, and drive-through take-away outlets can tempt customers into making unhealthy purchases.

In addition to TV-led campaigns, LiveLighter® messages are strategically placed in the community at locations where access to convenient, unhealthy food and beverages is high, in order to intercept decision-making and discourage unhealthy purchases. For example, the ‘Junk Food’ campaign placed messages to avoid drive-throughs on billboards and bus stops near fast food outlets. The ‘Eat Brighter, LiveLighter®’ campaign, which centred around increasing the amount and variety of fruit and vegetables consumed, was delivered through secondary (non-TV) media channels (billboards, digital screens, public transport, social media, radio, magazines, in shopping centres and at community festivals), in order to target people at key points of purchase or when they are thinking about meal preparation.

### Strengths and limitations

A key strength of this evaluation is the collection of cross-sectional data from prior to the launch of the program in 2012 and after almost all mass media campaign waves, enabling monitoring of impacts both pre- and post-campaign, and between campaign phases. To our knowledge, this evaluation provides the longest temporal coverage of an adult-targeted mass media campaign for healthy weight and lifestyle promotion and education in the world, and evidence of the longer term impacts on knowledge, intentions and behaviours associated with a sustained campaign of this type have not been reported elsewhere.

Limitations of the evaluation include the low response rates received for the cross-sectional surveys, which may have negatively impacted the validity and reliability of the results by contributing to non-response bias within the sample. Self-reported measures of health behaviours and body weight may be prone to social acceptability bias. Short questions to assess health behaviours are routinely used in national surveys and the baseline results in this study are consistent with Australian survey data [70]. While some studies assessing other campaigns have used alternative data sources for evaluation, such as product sales data, these were unavailable for evaluation in WA [71, 72].

The use of a repeated cross-sectional study design is a limitation of this study [38]. Cohort study designs that use a non-program comparison location are generally regarded as being more robust methodologies for evaluating public health campaigns than cross-sectional and uncontrolled designs. Several cohort studies have previously reported evidence for the effectiveness of the LiveLighter® program in other Australian jurisdictions [38, 40]. This includes studies demonstrating that the effects associated with the LiveLighter® program observed in states exposed to the program are not replicated in comparison states, adding further credence to the program as a driver of these positive changes [38, 40]. However, while the impact of the LiveLighter® program has been demonstrated in a number of Australian states, this impact may not generalise to other countries.

Isolating the extent to which a public health program impacts community-level outcomes can be challenging [19, 20, 69] given that multiple environmental factors are capable of shaping behaviour, including shifts in the social discourse about health particularly the social media phenomenon of the ‘wellness movement’, and measuring these factors can be challenging. However, the LiveLighter® campaign was the only government-funded healthy weight and lifestyle education and promotion program delivered in the state of WA during the study period. The high levels of awareness of the campaign in WA, along with this and other evaluations of the LiveLighter® campaign using non-exposed populations provide solid evidence for the campaign’s effectiveness.

Given the post-campaign positive changes in near-term outcomes including knowledge, intentions, and behaviours, there is a strong argument that the LiveLighter® program will contribute to long-term health outcomes and likely economic benefits [37, 73]. In 2020, an economic evaluation for an average LiveLighter® campaign used a meta-analysis of dietary changes reported in a cohort design evaluation study, to model changes in energy intake and weight in the WA population aged between 25 and 49 years over one year. The evaluation estimated the impact of these changes in energy intake and weight on incidence of obesity-related diseases and associated health care cost-savings over the lifetime of the modelled WA population. The evaluation found that the LiveLighter® program in WA produced both cost-savings and health benefits and was deemed to be highly cost-effective [73].

## Conclusion

The LiveLighter® campaign is associated with improvements in knowledge of the serious health consequences associated with overweight and obesity and with increased intentions and behaviours as they relate to following a healthy diet. These findings support the use of sustained, well-designed public health education programs that include mass media campaigns to promote healthy weight and lifestyles, as part of a comprehensive range of policies and programs to improve population dietary habits and reduce the burden from chronic disease. The evidence published to date provides a strong rationale for ongoing investment in the continued implementation of the program [74].

### Electronic supplementary material

Below is the link to the electronic supplementary material.

Additional File 1: Campaign Material– Behind a grabbable gut is cancer-causing toxic fat

Additional File 2: Campaign Material– Sugar your body doesn’t need gets turned into toxic fat

Additional File 3: Campaign Material– Had a gutful of junk food?

Additional File 4: Campaign Material– Sugary drinks are a rotten choice

Additional File 5: Campaign Material– You sure you want fries with that?

## Data Availability

The data used in this study were collected as part of a confidential service agreement between WA Department of Health, the Heart Foundation (WA) and Cancer Council WA and are not publicly available.

## Abbreviations

ADG: Australian Dietary Guidelines
AUD: Australian Dollar
BMI: Body Mass Index
CATI: Computer Assisted Telephone Interviewing
OECD: Organisation for Economic Cooperation and Development
RDD: Random Digit Dialling
SSB: Sugar-sweetened Beverage
TARP: Target Audience Rating Point
WA: Western Australia
WHO: World Health Organization
