# Supplementary figures and images for: Evaluation of the population-level impacts of the LiveLighter® obesity prevention campaign from 2012 to 2019 based on serial cross-sectional surveys
Source: BMC Public Health. 2024 Apr 12;24:1016. doi: 10.1186/s12889-024-18462-5 (PMC11010377; doi:10.1186/s12889-024-18462-5)

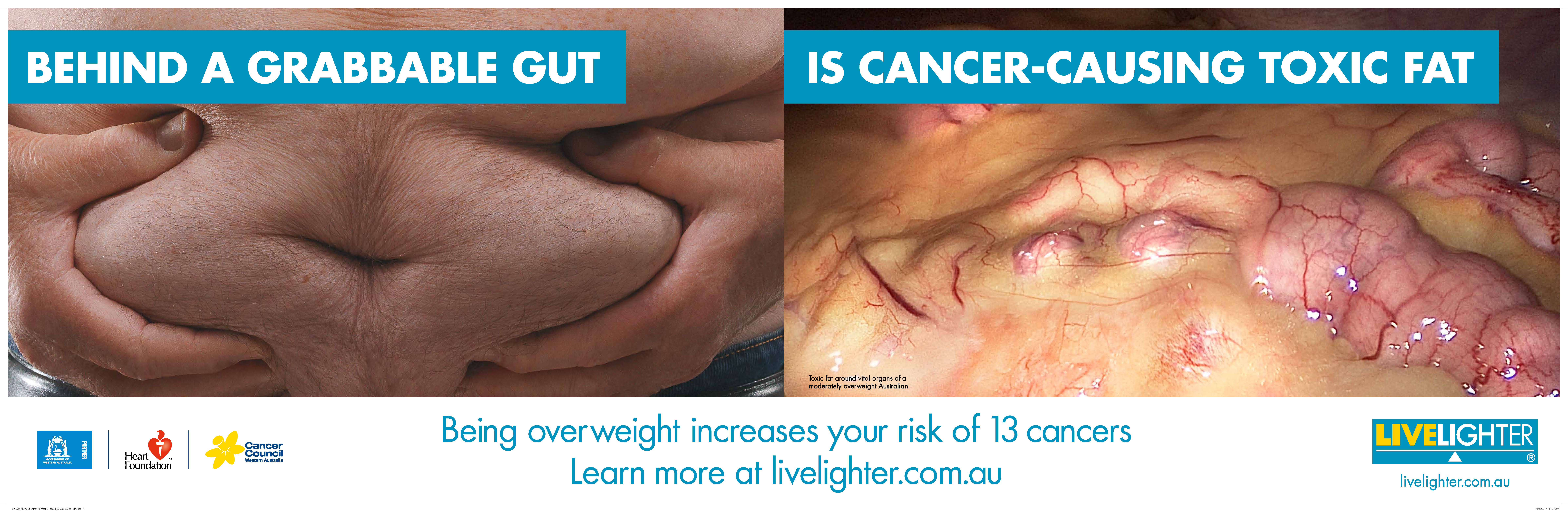

Supplement: Supplementary file 1 — Additional File 1: Campaign Material– Behind a grabbable gut is cancer-causing toxic fat [file 12889_2024_18462_MOESM1_ESM.jpg]

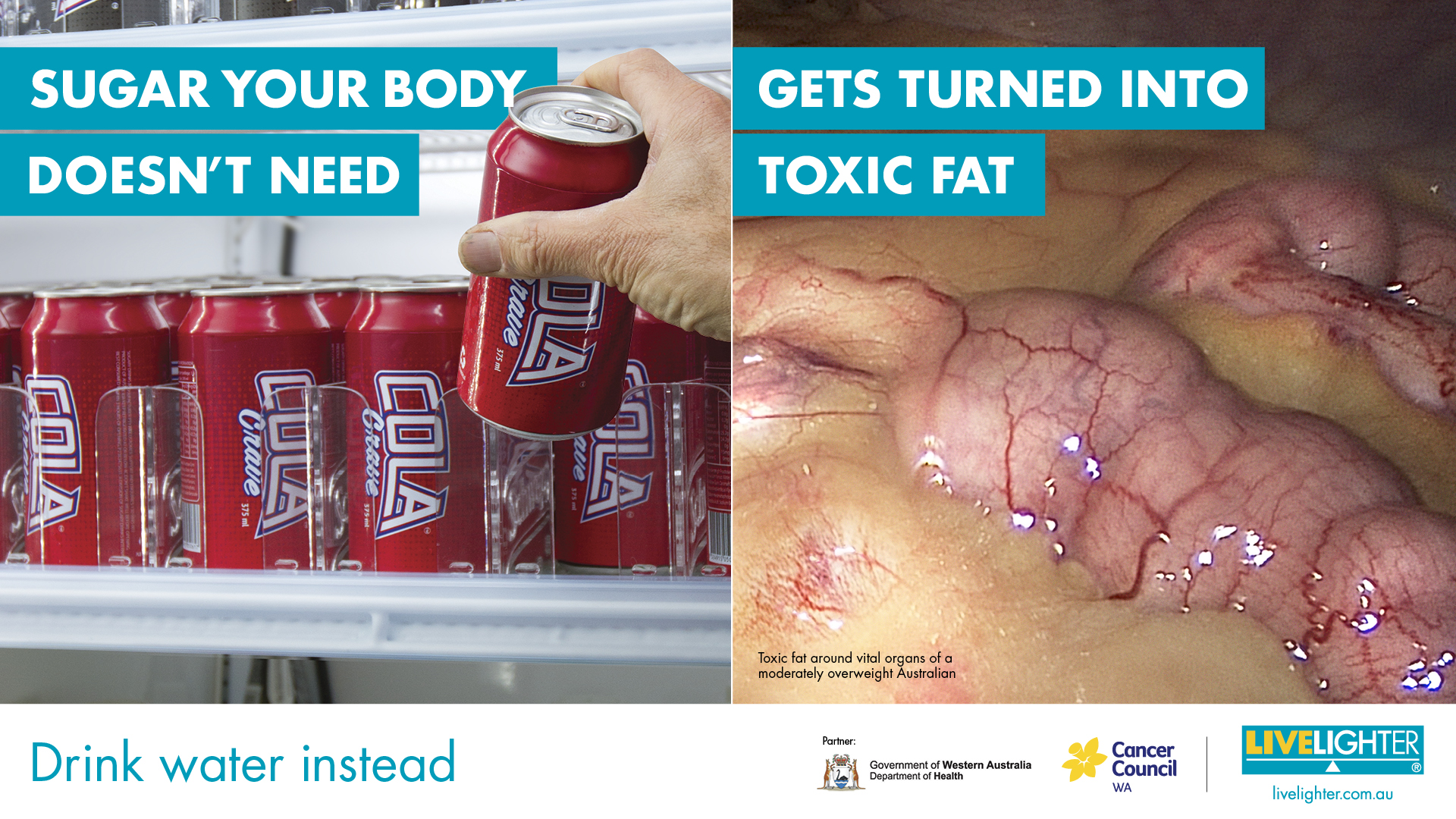

Supplement: Supplementary file 2 — Additional File 2: Campaign Material– Sugar your body doesn’t need gets turned into toxic fat [file 12889_2024_18462_MOESM2_ESM.jpg]

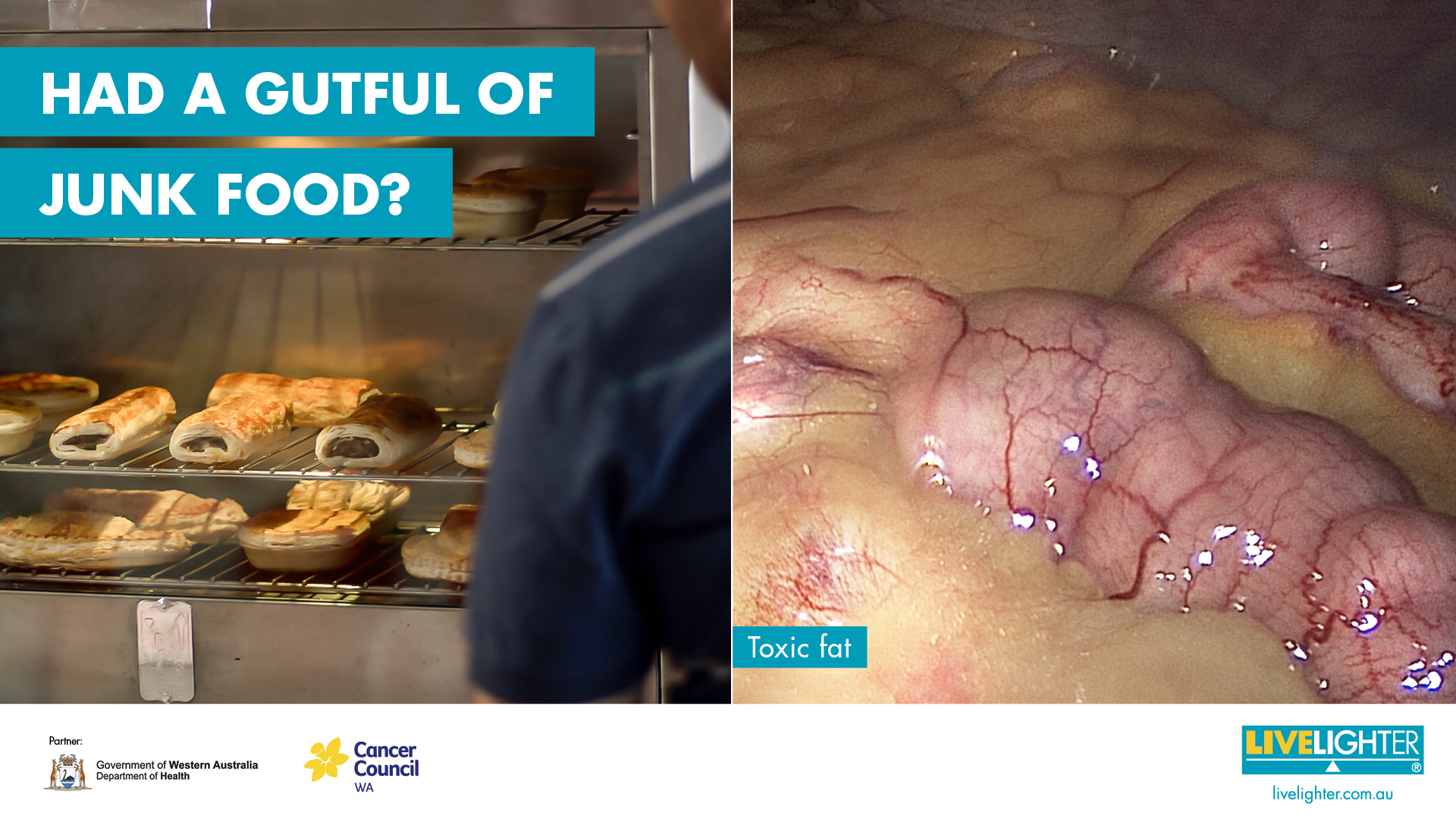

Supplement: Supplementary file 3 — Additional File 3: Campaign Material– Had a gutful of junk food? [file 12889_2024_18462_MOESM3_ESM.jpg]

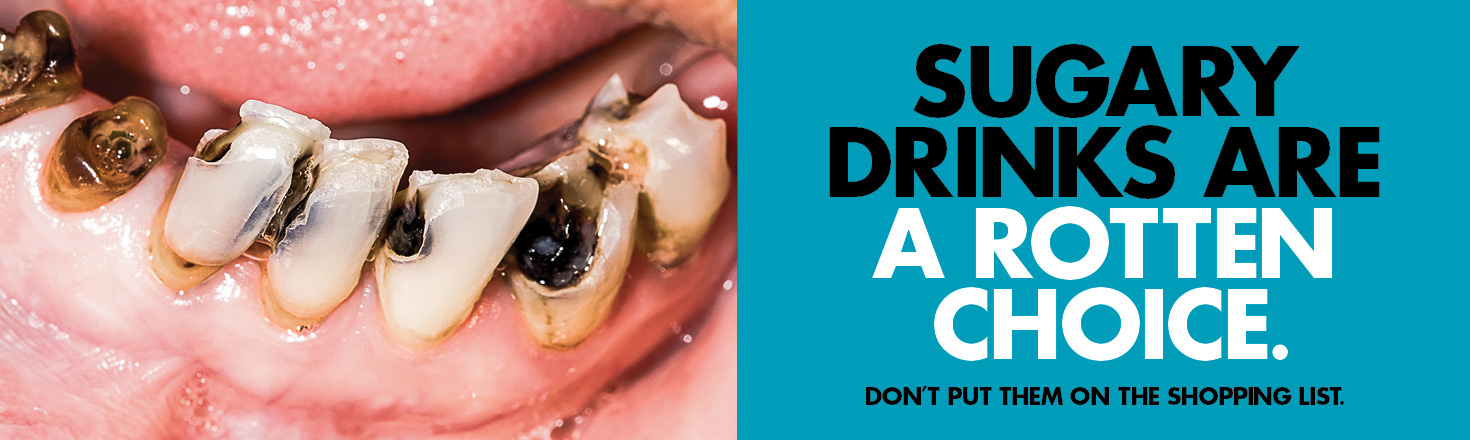

Supplement: Supplementary file 4 — Additional File 4: Campaign Material– Sugary drinks are a rotten choice [file 12889_2024_18462_MOESM4_ESM.jpg]

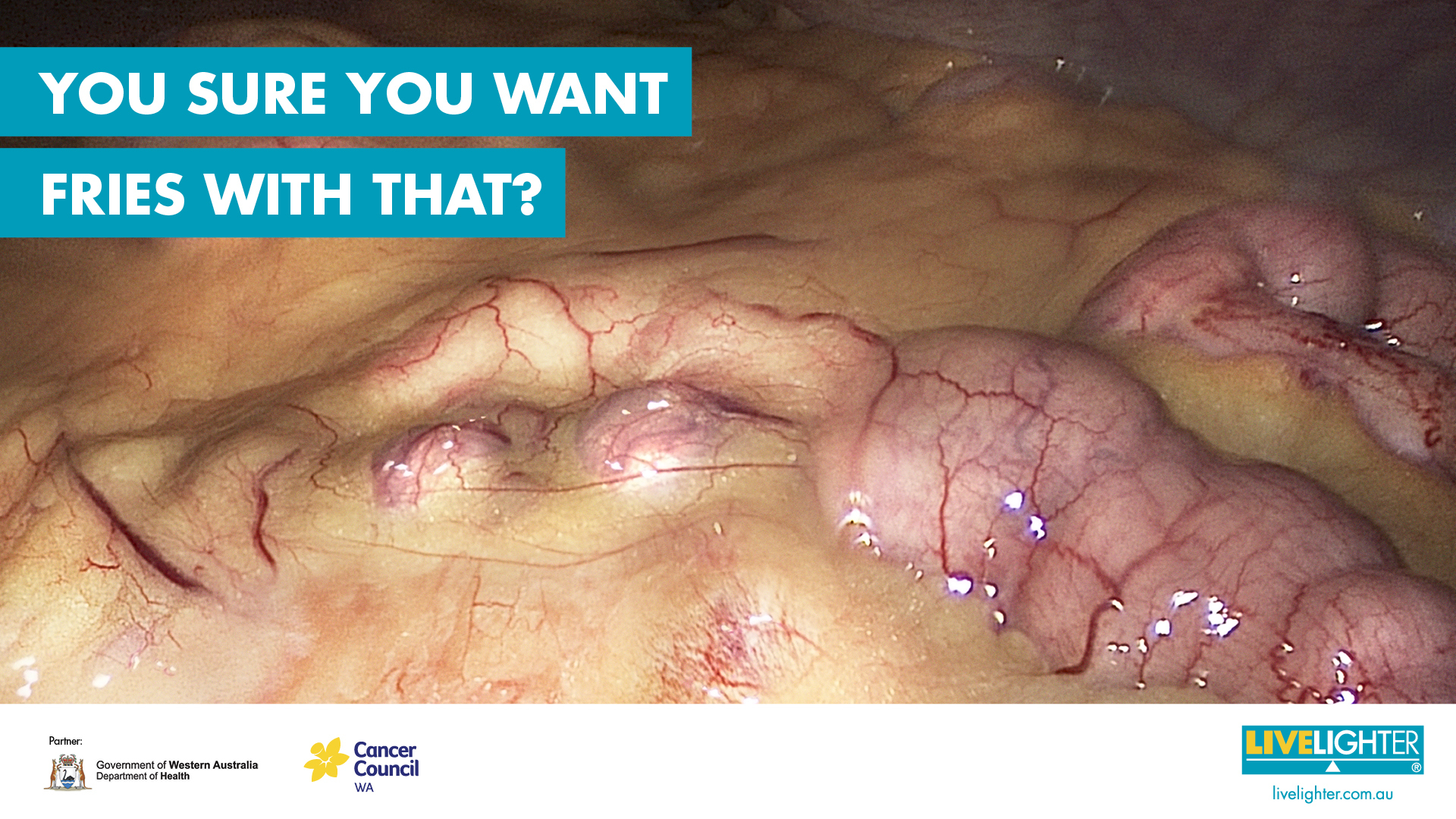

Supplement: Supplementary file 5 — Additional File 5: Campaign Material– You sure you want fries with that? [file 12889_2024_18462_MOESM5_ESM.jpeg]
